# *Experiments as Code* and its application to VR studies in human-building interaction

**DOI:** 10.1038/s41598-024-60791-3

**Published:** 2024-04-30

**Authors:** Leonel Aguilar, Michal Gath-Morad, Jascha Grübel, Jasper Ermatinger, Hantao Zhao, Stefan Wehrli, Robert W. Sumner, Ce Zhang, Dirk Helbing, Christoph Hölscher

**Affiliations:** 1 Chair of Cognitive Science, ETH Zürich, Zurich, Switzerland; 2 Data Science, Systems and Services Group, ETH Zürich, Zurich, Switzerland; 3 Cambridge Cognitive Architecture, University of Cambridge, Cambridge, UK; 4 Geo-information Science and Remote Sensing Laboratory, Wageningen University, Wageningen, The Netherlands; 5 Game Technology Center, ETH Zürich, Zurich, Switzerland; 6 Visual Computing Group, Harvard University, Cambridge, USA; 7 Center for Sustainable Future Mobility, ETH Zürich, Zurich, Switzerland; 8 Geoinformation Engineering Group, ETH Zürich, Zurich, Switzerland; 9 Decision Science Laboratory, ETH Zürich, Zurich, Switzerland; 10 Chair of Computational Social Science, ETH Zr̈ich, Zurich, Switzerland; 11 School of Cyber Science and Engineering, Southeast University, Nanjing, China; 12 Purple Mountain Laboratories, Nanjing, China

**Keywords:** Psychology and behaviour, Civil engineering

## Abstract

Experiments as Code (ExaC) is a concept for reproducible, auditable, debuggable, reusable, & scalable experiments. Experiments are a crucial tool to understand Human-Building Interactions (HBI) and build a coherent theory around it. However, a common concern for experiments is their auditability and reproducibility. Experiments are usually designed, provisioned, managed, and analyzed by diverse teams of specialists (e.g., researchers, technicians, engineers) and may require many resources (e.g., cloud infrastructure, specialized equipment). Although researchers strive to document experiments accurately, this process is often lacking. Consequently, it is difficult to reproduce these experiments. Moreover, when it is necessary to create a similar experiment, the “wheel is very often reinvented”. It appears easier to start from scratch than trying to reuse existing work. Thus valuable embedded best practices and previous experiences are lost. In behavioral studies, such as in HBI, this has contributed to the reproducibility crisis. To tackle these challenges, we propose the ExaC paradigm, which not only documents the whole experiment, but additionally provides the automation code to provision, deploy, manage, and analyze the experiment. To this end, we define the ExaC concept, provide a taxonomy for the components of a practical implementation, and provide a proof of concept with an HBI desktop VR experiment that demonstrates the benefits of its “as code” representation, that is, reproducibility, auditability, debuggability, reusability, & scalability.

## Introduction

Human-Building Interaction (HBI) is a field dedicated to understanding how humans and buildings co-exist and influence each other^1^. HBI researchers often conduct behavioral experiments in existing buildings to gain insight into this dynamic relationship. However, these experiments face significant challenges, including privacy concerns, implementation difficulties, and lack of compliance^2^.

To overcome these challenges and increase the degree of internal validity, HBI researchers are increasingly turning to Virtual Reality (VR) to conduct behavioral experiments, allowing cost-effective pre-occupancy evaluations^3–7^. Behavioral experiments in VR are composed of more elements than just the content of the experiment, ranging from the development of tasks, procedures, information, and documentation. Moreover, VR experiments should also include the experimentation process^8^ that describes how to conduct an experiment rather than purely describing the outcome. Experimentation includes all steps from preregistration to the acquisition of participants and monitoring the status of the infrastructure. Based on best practices, we classify these elements as components in groups and subgroups under a newly proposed six-pillar taxonomy (see Fig. 2). These pillars cover the essential aspects of an experiment by subdividing it into fundamental groups: documentation, infrastructure & environment, data collection, data assembly, data analysis, and management.

Typically, VR behavioral experiments in HBI are inspired by an industry standard procedure for testing user interfaces in computer programs (HCI) with small user studies (4–6 participants)^9^. Whereas user studies in user experience research test a user interface for a computer program with a specific task, HBI requires a more complex setup entailing importing virtual building models into a game engine and exploring it. Ad hoc explorations of the buildings similar to a user study may not suffice to investigate the building as even for user studies the 5-participants rule has long been discredited^9^. Furthermore, since there are no mechanisms to easily reproduce or audit these ad hoc experiments, it is difficult to assess their validity. At the same time, critical and often expensive design decisions are made based on anecdotal findings from these experiments.

This difficulty in validating VR experiments in HBI is closely related to the crisis of scientific reproducibility in experimental behavioral research in general ^10–13^. Many efforts to replicate have failed to reproduce the original findings, raising concerns about the reliability of the research results^10–13^. This reproducibility crisis is exacerbated by the informal sharing of experiment details, often conveyed verbally, which can lead to missing information and hinder replication efforts^14^.

Current efforts to achieve reproducibility have improved the situation such that enough information is provided to “understand” the experiment. However, understanding alone may not be sufficient for re-implementing the experiment from scratch. Subtle details may be implemented differently, leading to different outcomes^15^. In contrast, Preproducibility^16^, or scientific recipes, requires researchers to provide the complete set of elements and procedures necessary to replicate an experiment that “cannot with advantage be omitted”^17^. Numerous initiatives across various scientific disciplines aim to improve experimental research, enabling reusability and composability^18–23^. While several suggestions have been made to address the reproducibility crisis and improve experimental research in general^24–30^, a common framework is needed to implement and communicate these solutions effectively. Therefore, an actionable way of implementing the concept of preproducibility is necessary.

To address these grand challenges and at the same time improve the reproducibility of VR experiments in HBI, we propose the concept of “Experiments as Code” (ExaC) as a paradigm to glue together and communicate best experimental practices. This approach draws inspiration from cloud computing, that focuses on the delivery of computing services over the internet, providing on-demand access to resources like storage and processing power, eliminating the need for organizations to manage their own physical infrastructure.

First, there has been a notable shift in software development practices, where various aspects of systems and processes are now represented as code. The idea is that instead of manually configuring and managing these systems by setting all possible parameters, these parameters are defined and controlled through code scripts. The practice focuses especially on putting into code steps that previously humans did (like copying data, starting a program, installing a program, and checking the internet connection). For example, in the realm of software infrastructure, there is a practice known as “Infrastructure as Code” (IaC). It involves the use of code to describe and automate the setup of infrastructure components, such as servers and databases. In the context of our work, “Experiments as Code” (ExaC) follows the same principle by representing all elements of an experiment and its experimentation, including its procedures and documentation, in a structured and executable code format. Thereby, this approach enables automation and reproducibility of all steps of the experimentation, similar to how IaC automates infrastructure management.

Second, the “as a Service” revolution has transformed the way tasks are solved over the Internet. Instead of doing a task yourself, a service provides the task results by encompassing a wide range of functionalities, from cloud computing and storage to software applications and data analytics. Services are often provided on a subscription basis (servicising)^31,32^ and have become integral to modern technology solutions. In the context of ExaC, we leverage these principles with experiment-related services, thereby making it easier for researchers to access and use experiment components without requiring in-depth technical expertise.

In essence, ExaC means representing every aspect of an experiment, from its components and procedures to documentation, in a structured and executable code format^16^. This code-based representation makes it easier to reproduce experiments, understand their mechanics, and ensure transparency. It also brings benefits such as auditability, debuggability, reusability, and scalability to the experimental process^23^.

In this paper, we explore the ExaC paradigm and its potential to enhance the reproducibility of VR experiments in HBI. We will define ExaC explicitly and show its implementation in a desktop VR experiment^7,33^ for the HBI domain.

The proof of concept provides a code representation of components in the six pillar taxonomy for this experiment, see Fig. 2, that enables the automation of the instantiation of everything necessary for running the experiment and providing documentation for it, see Fig. 5. Several standard technologies, e.g., Docker, Terraform, Python, Golang, jupyter notebooks, MTurk, S3 Object storage are used, details on the implementation of every component can be found in the “Methods” Section. The experiment consists of participants finding their way to different targets within a building. The experiment evaluates the effect of two architectural variations on the wayfinding performance measured in terms of distance walked to find the targets. ‘Treatment A’ adds Atria to the base building and ‘Treatment B’ modifies the staircase shafts with transparent glass, see Fig.  4. The advantages of an ‘as code’ representation of the experiment and its experimentation process are evaluated by instantiating the experiment several times, auditing, debugging, and reusing the experiment components, and by analyzing the user pool that accessed the experiment.

The main contributions of this paper are threefold. First, we contribute towards establishing an ExaC paradigm by providing a definition and demonstrating it with the full experimental pipeline of a desktop VR behavioral HBI experiment. Second, we provide an open source proof of concept implementation of this “Experiment as Code” paradigm, to scaffold data collection in our online desktop VR experiment. This enables third parties to audit and reuse our experimental design and demonstrates the benefits of the ExaC representation. Third, we apply the lens of ExaC to notable VR frameworks and provide a detailed comparison and review of these, emphasizing their capacity and shortcomings to support experiments, experimentation, and Preproducibility.

### Concept

Behavioral experiments in VR can be composed of several elements (e.g., tasks, procedures, information, and documentation) beyond the experiment content. They can span from preregistration over the acquisition of participants to the monitoring of the infrastructure status. We collect experimental elements we have observed in our interdisciplinary experience in behavioral experiments in HBI and identify gaps in how the current VR Frameworks support them, see Table 3). We classify these elements as components in groups and subgroups under the proposed six-pillar taxonomy (see Fig. 2). The pillars cover the essential aspects of an experiment by subdividing it into groups: documentation, infrastructure & environment, data collection, data assembly, data analysis, and management. We collect and exemplify these common components required to enable reproducibility. The exemplified components may vary in their classification and extent depending on the details of their implementation and experiment requirements; e.g., sensitive data anonymization could be desirable to occur at collection time. Although some components of each pillar are strictly required to produce an experiment, others are optional but desired to improve reproducibility.

We base our definition of ExaC on the idea of preproducibility^16^, but instead of providing *scientific recipes* we aim at providing *automated and digitally documented scientific recipes* inspired by the advances in cloud computing. Thus, we define the ExaC paradigm as providing imperative, or declarative automation code and digital documentation to the components of the respective essential pillars.

**Figure 1:**
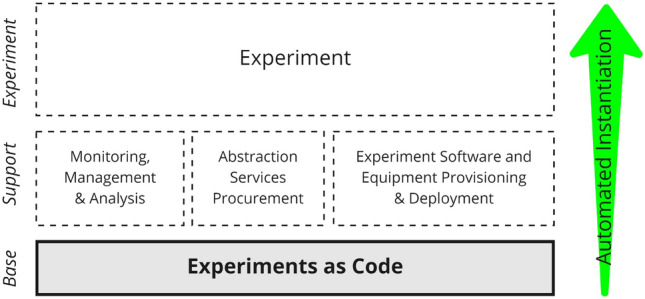
ExaC paradigm application scheme: The paradigm serves as the Base image to instantiate the Support (infrastructure and services) and then conduct the Experiment itself. The ExaC (gray box) covers the six-pillar taxonomy of behavioral experimentation (see Fig. 2).

We borrow the imaging concept from Environments as Code. An image or snapshot of ‘how a run-time environment should look like’ serves as a specification of how actual containers are deployed. The static ExaC codebase serves as the image (see Fig. 1, Base). The whole live experimental setup can be automatically instantiated from the image including the supporting components and the components building the experiment itself. This entails but is not limited to provisioning the right hardware, deploying the software stack, instantiating abstraction interface services, acquiring participants, hardware monitoring services, serving the content to participants, and ultimately collecting the experiment data.

The ExaC paradigm is rooted in three larger developments of the last decade. First, the rise of the field of *meta-science*^34^, second are initiatives to overcome the reproducibility crisis, and third are developments in cloud computing such as the “___ as Code” and “___ as a Service” revolutions.

There are several initiatives that tackle challenges impacting directly reproducibility. For instance, there is an indexing service that specializes in indexing papers accompanied by code^18^. Another initiative provides multidisciplinary, collaborative, and reproducible data pipelines^19^. There are also modular ecosystems to improve machine learning reusability by managing and automating the entire lifecycle of machine learning application development life-cycle with reusable models and components^20^. Some disciplines have pushed even further, creating domain specific standards^21^ and ontological databases^22^ that enable more efficient and systematic knowledge extraction. Moreover, publishers are now encouraging the publication of protocols in specialized services such as protocols.io, where protocols are converted to modifiable and reusable checklists.

Tightly interrelated with reproducibility are the notions of auditability, debuggability, reusability, & scalability. We define auditability as providing a transparent and comprehensive view into the experiments’ mechanics, tools, and components. Thereby, any third party is able to evaluate the software^35^ and by extension the experiment’s reproducibility. We define debuggability as the means to identify and modify aspects of the experiment that affect its reproducibility^36^. We define reusability as the ability to reuse parts or variations of an existing software to create a new one^37^ and in our context software extends to experiments. Here we can exploit the benefits of open source software development in science,^38^. These three are ubiquitous tasks in open-source software development and as such are enabled through the ExaC representation by inheriting the same good practices. Finally, in systems scalability refers to the ability to either expand a system in one dimension (structural) or increase in the amount of activity (load) the system receives^39^. In the context of experiments, this refers to the ability to increase the sample size either through repetition (horizontal) or the use of larger participant pools (vertical), e.g., crowd-sourced and remote experiments.

In cloud computing, the complexities and requirements of current web services have pushed advances and paradigm shifts such as the “___ as Code” and “___ as a Service” revolutions. Paradigms such as “Infrastructure as Code” (IaC; e.g., Terraform, AWS Cloud formation), “Environments as Code” (e.g., Vagrant, Docker), “Configuration as Code” (e.g., Ansible, Puppet Chef), “Data Pipelines as Code” (e.g., Airflow) are now ubiquitous in web service development. There, a single service requires the deployment of hundreds to thousands of micro-services, their infrastructure to be dynamically provisioned, and their data to be continuously analyzed.

**Figure 2:**
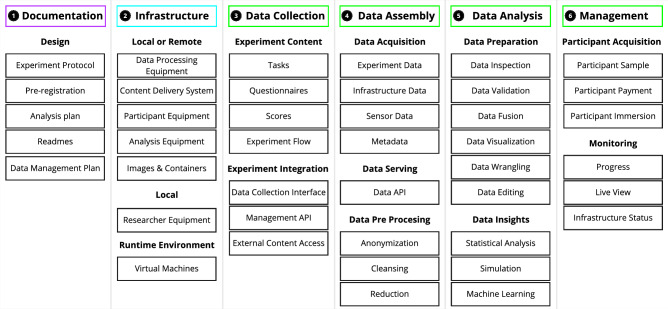
Six-pillar taxonomy of behavioral experimentation: (1) documentation, (2) infrastructure & environment, (3) data collection, (4) data assembly, (5) data analysis, (6) management. Each pillar consists of multiple essential tasks that need to be completed. Each pillar is distinctively represented in the ExaC paradigm as a block for the automation of an experiment.

For example, IaC allows storing and versioning the infrastructure and configuration requirements of complex web-services. These code representations have enabled simpler deployment and provisioning, rolling back to previous configurations, mirroring services, planning, scaling, and even debugging problems in the infrastructure provisioning. We believe that many of the features of the “___ as Code” movement and their tools could enhance experiments in general and VR experiments in particular.

Despite this substantial drive for improving the reproducibility of behavioral experiments, Preproducibility is still a challenge. However, the boom of “___ as Code” provides a foundation to implement Preproducibility and achieve reproducibility in behavioral experiments. To the best of our knowledge, the term “Experiment(s) as Code” has only been used in two research articles. First, in EnosStack^40^ that proposes a software stack composed of Python, Ansible, Docker, and their EnosLib library to enable reproducible experimental workflows. Second and similarly,^41^ identified the need for reproducible virtual labs and created a full platform. In contrast to these works, we define the ExaC paradigm and scaffold it onto a taxonomy describing how to conduct VR experiments as an implementation of Preproducibility. This involves combining a wide variety of approaches from “___ as Code” with VR frameworks and other complementary software components of the experiment. Moreover, the components are only loosely coupled allowing them to be exchanged as services evolve.

## Results

**Figure 3:**
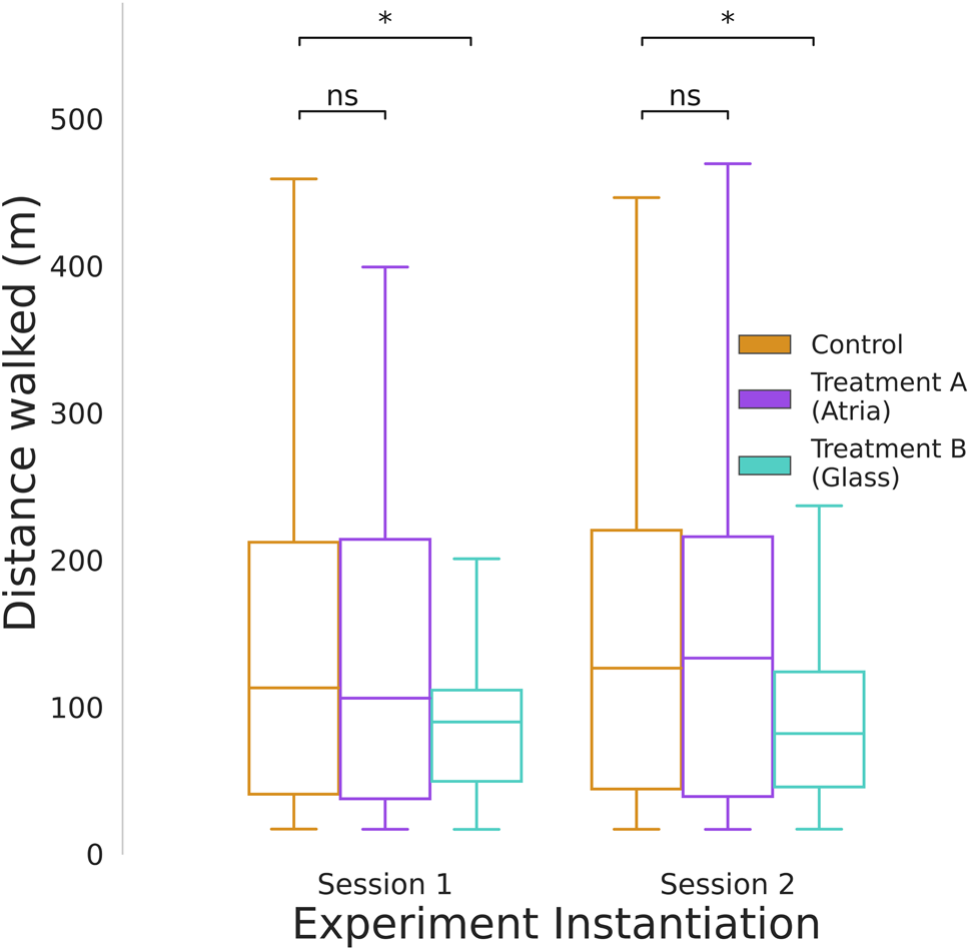
The same pattern of results is shown by the two complete instantiations of the experiment, i.e., improvement in walking performance for treatment B, the glass condition. Session 1 (n = 275), Session 2 (n = 495). For an in-depth data analysis of the experimental results, see^33^.

**Reproducibility** is assessed by independently evaluating the results of the two complete instantiations (Session 1 and Session 2) with the Linear Mixed Effect Model: $\text{ DistanceWalked } \sim \text{ Treatment } + ( 1 | \text{ Participant})$, see Table 1. Table 1 presents at the top panes the model diagnostics statistics for each session, e.g., that the models converged, the number of observations, group sizes, log-likelyhood. This shows for example that both models are healthy, i.e., converged. At the bottom panes the modeling results, i.e., mean distance walked in the base condition, i.e., intercept (control), as well as the effect size of the different treatments, their p-values, and confidence intervals. Figure 3 shows that in both instantiations of the experiment, the same pattern of results can be observed, treatment A (Atria) has no significant effect, while treatment B (Glass) provided a significant ($p<0.05$) performance improvement. It has to be noted that the reproducibility of this experiment can only be assessed with respect to the specific claims made by the original paper^33^ and the statistics supporting it. In this case, the claim is a significant increase in walking performance substantiated by mixed-effects analysis and the p-value of the factor for treatment B, which holds true.

**Table 1:** Analysis results of the two instantiations of the experiment using a linear mixed effect model.

(a) ﻿Session 1						
Model:	MixedLM	Dependent Variable:	DistanceWalked			
No. Observations:	275	Method:	REML			
No. Groups:	46	Scale:	20131.3268			
Min. group size:	5	Log-Likelihood:	− 1740.4921			
Max. group size:	6	Converged:	Yes			
Mean group size:	6.0					

	Coef.	Std.Err.	z	P$> |$z|	[0.025	0.975]
Intercept (Control)	148.717	15.040	9.888	0.000	119.239	178.195
Treatment A	4.446	21.211	0.210	0.834	− 37.126	46.018
**Treatment B**	− 50.988	20.878	− 2.442	**0.015**	− 91.909	-10.067
Group Var	0.000	10.558				

(b) ﻿Session 2						
Model:	MixedLM	Dependent Variable:	DistanceWalked			
No. Observations:	495	Method:	REML			
No. Groups:	83	Scale:	25518.3730			
Min. group size:	4	Log-Likelihood:	-3201.8899			
Max. group size:	6	Converged:	Yes			
Mean group size:	6.0					

	Coef.	Std.Err.	z	P$> |$z|	[0.025	0.975]
Intercept (Control)	177.752	11.387	15.611	0.000	155.434	200.069
Treatment A	− 22.157	15.961	− 1.388	0.165	− 53.439	9.126
**Treatment B**	− 78.186	20.028	− 3.904	**0.000**	− 117.440	− 38.931
Group Var	0.000	7.662				

Significant values are in [bold].

**Figure 4:**
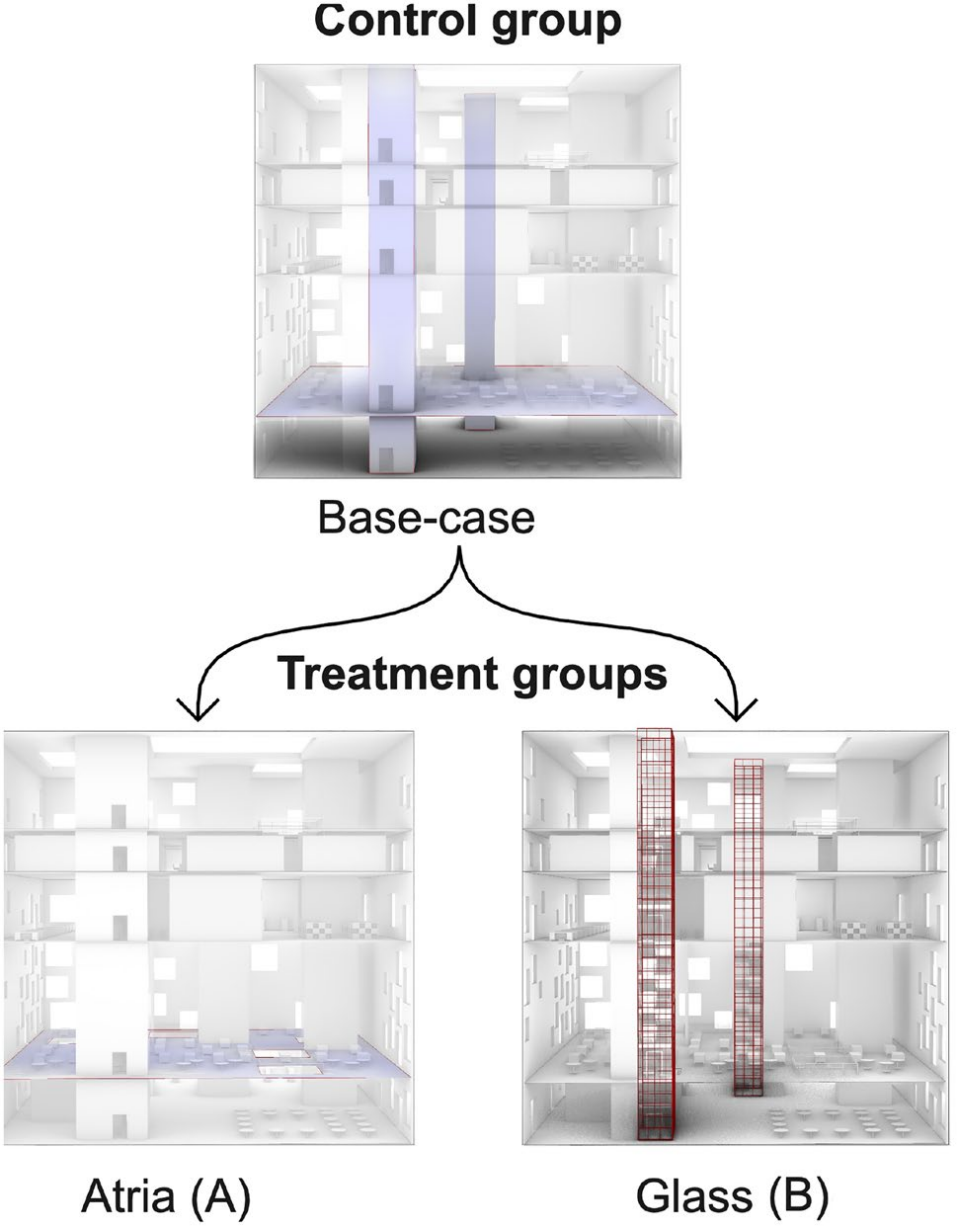
Base case and the two treatment conditions. Treatment A, the addition of atria in the floor, Treatment B, making the stair shafts transparent with glass.

**Auditability, Debuggability, & Reusability** are shown through a simulated third-party auditing of the experiment and describing bugs found while using the code. This is documented in a GitHub issue ‘Example 1 - Bug fix’, see Fig. S1 in the Supplementary Information. In response to this GitHub issue, the maintainer identifies the cause of the bug and creates a new version reusing most of the code. Additionally, the GitHub issue ‘Example 2 - Reuse’, shows a simulated interaction with a researcher reusing the components of the original experiment for an online version of another desktop VR wayfinding experiment, i.e.^42^, see Fig. S2 in the Supplementary Information. Here, the researcher adds the experiment assets, i.e., the building and building variations, and implements the experiment mechanics, e.g., door frames changing in color. These modifications are limited to the data collection module, while all the other modules are reused without changes. All GitHub issues presented here as examples are linked with their respective code. Interested third parties are encouraged to check the repository Issues section in addition to auditing and forking the repository.

**Figure 5:**
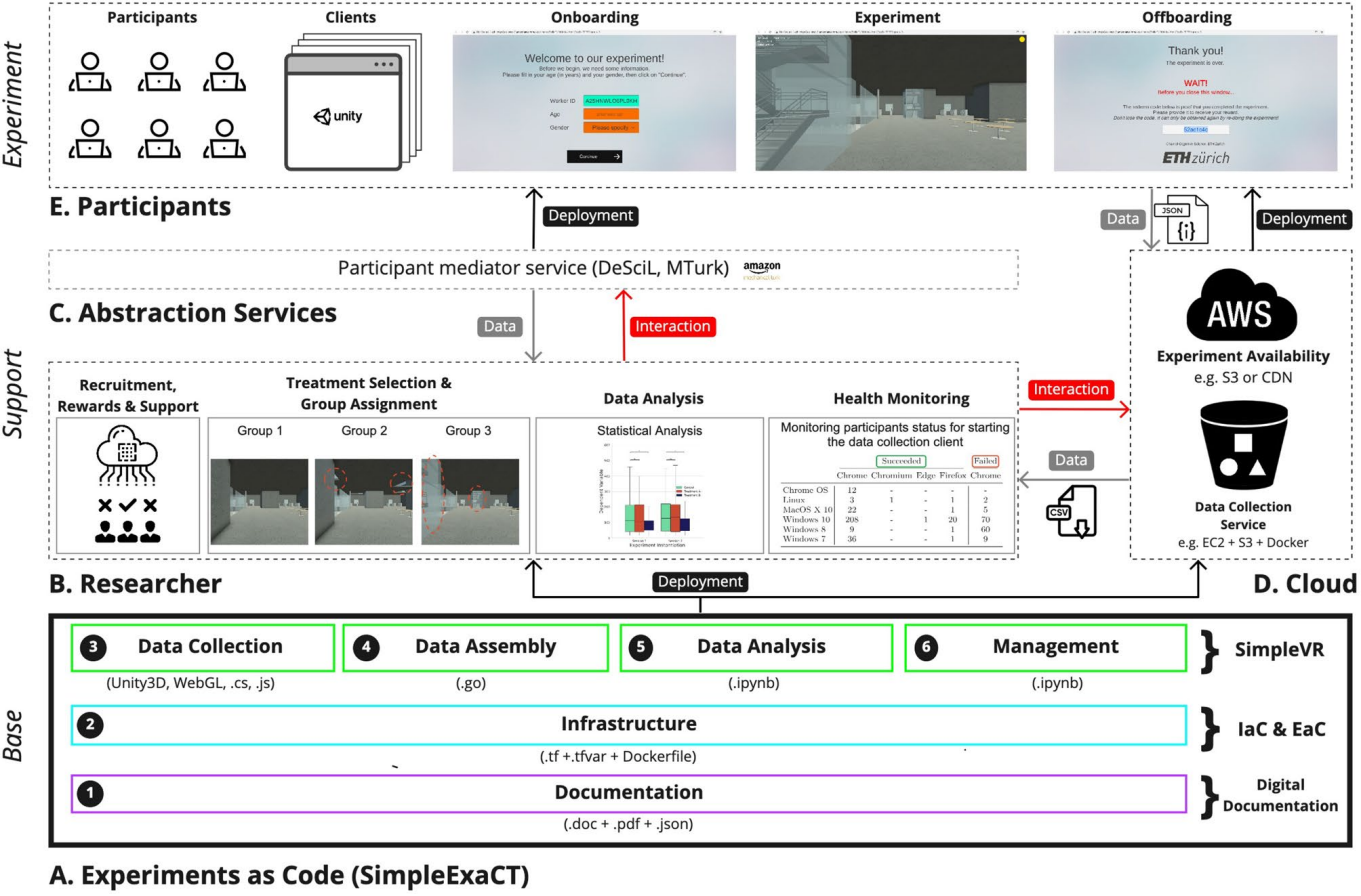
Overview of the ExaC implementation and instantiation for the purpose of our case-study experiment (see section “Demonstrative Case Study Design”). The illustration integrates the ExaC implementation (Base), the instantiated services and infrastructure (Support), and the data collection client (Experiment).

**Scalability** is evaluated by the ease of recruiting participants in the different instantiations of the experiment and investigating the access to the experiment beyond the 149 participants that successfully took part in the experiment by reviewing the raw accesses to the experiment. This includes all the interactions with the experimental data collection (i.e., MTurkers that clicked on the experiment link, see Fig. 5, E). In total, 462 participants accessed the experiment, 316 (68%) had computers that were deemed capable of displaying the experiment, while the remaining 146 (32%) were not. Of the participants starting the experiment, 149 (47%) participants also completed the experiment. The participants’ inferred Operating System and browsers are reported in Table 2.

**Table 2:** Excerpt from the participant monitoring.

	Succeeded			Failed
	Chrome	Firefox	Other	Chrome
Chrome OS	12	–	–	–
Linux	3	1	1	2
MacOS X 10	22	1	–	5
Windows 10	208	20	1	70
Windows 8	9	1	–	60
Windows 7	36	1	–	9
Total	290	24	2	146

The operating system and browser are inferred from the self reported browser user agents. The client was successfully rendered on 316 computers and failed to render on 146 computers.

## Discussion

Research needs to address the validity of their claims or explain deviations from previous results like behavioral drifts or changes in implicit experimental conditions^15^.

Behavioral experiments in VR often require time-consuming setup and diverse expertise in specialized hardware (e.g., physiological sensors^43,44^) or infrastructure well beyond the capacity of smaller research groups and researchers^45,46^. This difficulty, coupled with the implicit information required to reproduce an experiment, has contributed to the reproducibility crisis across many disciplines. We believe that this crisis can be mitigated through the use of emergent technologies such as the ones behind the “___ as Code” and “___ as a Service” movements. The “___ as Code” technologies provide a tool-set for automation and reproducible software environments. Together with VR frameworks, this enables the implementation of the so far conceptual notion of ‘Preproducibility’. The “___ as a Service” technologies provide outsourcing and abstraction layers that can be used to interface with real world needs such as the recruiting and rewarding of participants.

To explore the applicability of “___ as Code” and “___ as a Service” to behavioral experiments in VR, we first define a taxonomy for behavioral studies in VR (see Fig. 2) to define a scope in which automation could and should occur. We argue that there is a need for such a taxonomy to create a common framework for shareable work and modularity that can enable the reuse of individual components. Second, we proposed a definition of the ExaC paradigm as providing declarative or imperative automation code and digital documentation that corresponds to each of the experiment taxonomy pillars (see Fig. 1). By publishing their code base, researchers offer concrete means of reproducing their research. Hence, enabling their experiments to be reproduced, audited, debugged, reused, and scaled by other researchers. Beyond replication, their research can then be systematically varied to uncover robustness and generalisability. Additionally, different experimental approaches could be adapted to the same experiment, thus enabling triangulation^29^. In triangulation, the same concept is tested with different approaches to ensure generalisability.

To elucidate the applicability of the ExaC paradigm to support Preproducibility in VR experiments, we present a proof-of-concept implementation of the ExaC and apply it in a VR case-study in HBI. The proof-of-concept implementation of ExaC consists of the digital documentation, the infrastructure & environment definitions.

Our proof-of-concept implementation of ExaC requires a plethora of technologies. This might seem counterproductive at first sight as it could require strong expertise to master. The technologies and software stacks range from Docker, Terraform, Jupyter-Lab, Unity, to WebAssembly, and WebGL. On top of that, they require using several programming languages, Golang, Python, JavaScript, and C#. To add to this complexity, it has to integrate services that serve as an abstract interface to the physical world, such as DeSciL and MTurk. However, they are some of the best technologies available for each specific task and there is currently no one-size-fits-all solution. The additional effort will be amortized by reusing ExaC code bases. Even though monolithic highly coupled software can provide a more homogeneous user experience, we believe it would be difficult for it to keep up with the rapid advancement of the technology. Furthermore, these loosely coupled modular components can be exchanged as technologies progress or as requirements change.

Beyond the fast-paced development of technology, the loose coupling actually reduces end-user complexity. End users are only required to change the components they work on, enabling them to consider all other components *black boxes*. In the context of VR, this means that using ExaC allows experimenters to focus on experiment content rather than any aspect of deployment and provisioning.

There remain many technical challenges to be tackled for more general ExaC implementations beyond the proof of concept for the case study. For example, interfaces for specialized local or remote hardware, and specific protocols for the composition, description and management of services in the context of experiments need to be developed.

We showcase the reproducibility of our proof-of-concept implementation by analyzing two complete instantiations of the HBI experiment. We show that the pattern of results remains the same, i.e., ‘Treatment A’ (Atria) displays a null effect while ‘Treatment B’ (Glass) displays a statistically significant improvement in comparison to the ‘Control’ unmodified building. We showcase the auditability, debuggability, and reusability of the experiment by performing a task common in open source projects, i.e., a simulated auditor opened a GitHub issue and provided a description of a bug and a fix request, while the repository maintainer identified the required changes in the code and developed that fix. Additionally, we show the implementation of a second experiment by changing the data collection module and reusing the rest of the components. We demonstrate the experiment scaling in two ways, by enabling several sessions of data collection (horizontal scaling) and by recruiting participants from a big remote participant pool in comparison to the usually smaller local participant pools (vertical scaling). Even though our VR experiment was graphically demanding and data-heavy, we reached 462 participants of whom the majority (68%) had computers that were deemed capable of running the desktop VR experiment. From this pool, 149 participants successfully completed our experiment. This showcases the scaling potential of this experiment.

Many experiments pose more challenging reproducibility requirements, e.g., interfacing with specialized local hardware, or behavioral response changes due to nuanced variations in the setup, sensors, or equipment. Researchers can document as ExaC the solutions required to successfully run the experiment, e.g., local server setup and hardware interfaces, sensor configuration, or specialized hardware setup. This would enable themselves and third parties to reuse the solutions and would make the nuances transparent. With an ExaC ecosystem, researchers could focus on the specifics of their experiments while reusing parts of existing and tested experimental setups.

Communicating scientific results requires enumerating, recording, and reporting those things that “cannot with advantage be omitted”^17^. Depending on the discipline, Preproducibility^16^ might require information about materials (including organisms and their care), instruments and procedures; experimental design; raw data at the instrument level; algorithms used to process the raw data; computational tools used in analyses, including any parameter settings or ad-hoc choices; code, processed data, and software build environments; or even analyses that were tried and abandoned.

To the best of our knowledge, the term “Experiment(s) as Code” has only been used in two research articles. First, in EnosStack^40^ that proposes a software stack composed of Python, Ansible, Docker, and their EnosLib library to enable reproducible experimental workflows. Second and similarly,^41^ identified the need for reproducible virtual labs and created a full platform. In contrast to these works, we define the ExaC paradigm and scaffold it on to a taxonomy describing how to conduct VR experiments as an implementation of Preproducibility. This entails combining a wide variety of approaches from “___ as Code” with VR frameworks and other complementary software components of the experiment. Moreover, the components are only loosely coupled, allowing them to be exchanged as services evolve.

In this paper, we provide a conceptual grounding for ExaC based on Preproducibility. We provide a taxonomy of the components for a practical implementation and provide a proof-of-concept implementation that showcases the reproducibility, auditability, debuggability, reusability, and scalability of an experiment implemented following the ExaC paradigm.

It is well-known that automation of social processes can also have undesirable implications^47^. These problems range from loss of creative freedoms to new forms of exploitative, or alienating labor. We would like to distance ourselves from these practices. We believe that ExaC based experiments can mitigate these problems by ensuring transparent and auditable interactions with a society where fair practices such as rewards based on decent wages are enforced. Additionally, we would like to make a few clarifications to avoid possible misunderstandings and inappropriate generalizations of the concept proposed in this paper.

We have proposed to code and automate certain experimental procedures for the sake of reproducibility and other related advantages. However, we do not want to suggest intrusive large-scale A/B testing on a societal scale. We rather strive to achieve higher experimental standards than in conventional A/B-testing, presupposing fully informed consent, informational self-determination, and proper ethical approval of detailed experimental settings by an independent ethics committee deciding according to state-of-the art international standards.

We further aim for experimental settings that are interesting and rewarding. This implies, in particular, fair pay, avoiding unnecessary dull tasks, and alienating work. Furthermore, we caution against scaling up the automated experimental procedures to scales involving considerably sized (sub)populations of people or industrial/commercial applications without additional considerations and safeguards. In particular, here we are not proposing an automation of society^47^.

The ExaC paradigm is applicable beyond HBI research using VR experiments if we can define consistent abstraction interfaces to non-virtual components. Going beyond outsourcing research to specialized groups and facilities, the world advances with services that provide additional layers of abstraction. Such services allow us to interface with nonvirtual components (e.g., MTurk, DeSciL). Moreover, the ability to interface with reality from virtual worlds would enable innovative experiments based on virtual components, e.g., digital twins^48–50^, enhanced ABMs and multi-agent systems^51–60^ and medical applications^61^. In combination with the ExaC paradigm, it becomes possible to define reproducible virtual(ised) and mixed-virtual experiments in the real world.

There is a great potential in extending the ecosystem of “___ as Code” and “___ as a Service” to research facilities. Complex experiments in physics, chemistry, biology, and engineering could be transformed into a specification within the ExaC paradigm. This specification could be automatically sent to specialized facilities possessing unique equipment and domain knowledge of the best practices, ethical requirements, and efficient procedures. However, there are clear limitations as to how far this interfacing with non-virtual components can reach. For example, there could be sensitive interventions that could be dangerous or unethical to automate. Nonetheless, when the ExaC paradigm can be applied, it contributes to the democratization and improvement of the quality of research.

The ExaC paradigm and implementation could also provide an answer to challenging questions about reproducibility. For example, the inability to reproduce certain results in behavioral science could potentially be due to behavioral drifts. The individuals participating in a study could represent a group that has gradually changed its behavioral patterns due to societal, technological, environmental, ecological, or economical changes. When a study is reproduced, the previous results no longer hold as the group has shifted from previous behavioral patterns. By providing automation code, it would be possible for stakeholders to easily and continuously reproduce experiments and consequently observe the behavioral drifts over longer periods and across participants’ compositions.

The ExaC paradigm can also provide answers beyond behavioral drift by improving the generalizability of research results. Reusing flawed experiments over and over again could encourage the prevalence and perpetuation of bad practices. To counteract this, we believe that the ExaC ecosystem would need to adopt, document, and introduce best practices. However, the evolutive aspect of ExaC, if widely adopted, should also on its own weed out bad experiments and replace them with better versions. Additionally and beyond replication, triangulation^29^ can be applied to systematically vary experiments to uncover the underlying fundamental principles. Given the ability to easily reuse existing experiments, triangulated versions can be produced to shed light on phenomena from a different perspective.

In the future, we plan to further develop our proof of concept, providing better user-friendliness, as currently the instantiation of new experiments is performed through the command line. Previous work on VR has already produced robust frameworks for data collection (e.g., EVE^62,63^). We would like to integrate these frameworks to provide improved participant management, but also tools to aid in the rapid and effective design of experiments and multi-user environments^63^. Additionally, we would like to improve the analysis and data management of experiments by integrating complete machine learning lifecycles^20^. Finally, we want to extend the proposed taxonomy to more complete ontological relations^64,65^ and follow best practices in their design^66^. Combining all of these ideas could enable the creation of robust knowledge databases containing ExaC experiments and results.

In conclusion, we believe that the ExaC paradigm has great potential to enhance cooperation, enable equitable access to research resources, and underpin research in VR, especially for HBI. Moreover, we built a proof of concept based on the proposed taxonomy and demonstrated the possibility of implementing Preproducibility in a scalable online web-based VR behavioral experiment. Through this proof-of-concept ExaC implementation, we showcase reproducibility, auditability, debuggability, reusability, and scalability. The adoption of the ExaC paradigm can improve the quality of research across several domains, by providing reproducibility, auditability, debuggability, reusability, and scalability to experimental research.

## Methods

This paper explores the concept of “experiments as code” by examining what entails an experiment, how VR frameworks handle the experimental components, creating a practical implementation of the concept, and evaluating it in an HBI behavioral experiment. To this end, we first review the components in the six pillar taxonomy (documentation, infrastructure, data collection, data assembly, data analysis, and management, see Fig. 2). Second, we review common VR experimental frameworks used in HBI and compare them in the context of the six pillar taxonomy. Third, we describe how our proof-of-concept implementation of ExaC handles each of the components and the technologies it uses, see Fig. 5. Finally, we present the case study used to demonstrate the concept.

### Six-pillar taxonomy of behavioral experimentation

#### Documentation

Effective *documentation* forms the bedrock of any scientific experiment, providing a transparent and replicable framework for researchers. This pillar encompasses key elements crucial for experiment design and transparency. The Experiment Protocol outlines the step-by-step methodology, which should include a power analysis to determine the sample sizes and experiment design. The analysis plan establishes the foundation for the interpretation of the data. Readmes serve as comprehensive guides, offering insights into the experiment’s intricacies, and the Data Management Plan safeguards data integrity and accessibility. Pre-registration ensures a commitment to a predefined plan.

The documentation plays a pivotal role in delineating the intricacies of setting up and executing the experiment. Beyond its role in internal and external communication, the documentation serves as a foundation for validating a pilot study by comparing the anticipated outcomes with the actual results. Furthermore, it furnishes indispensable insights for understanding the acquired data (e.g., power analysis, protocols, task instructions), making sure the data comply with data management requirements like GDPR (e.g., data management plans), and the correct usage of the tools (e.g., READ.ME). It facilitates robust analyses necessary for composing scholarly papers. Although documentation is often informally crafted and often intended for internal use, particularly in communication between coauthors and experiment implementers, its significance becomes apparent when considering potential modifications to the experiment. In such cases, the documentation encapsulates invaluable information that proves instrumental in preserving the experiment’s integrity and facilitating future adaptations.

Documentation intended for external communication is now expected through preregistration (reviewed and unreviewed)^24^, but so far these have only been tentatively used in HBI VR studies. This pre-registration process requires elements such as a protocol, analysis plans, and data management plans. Data management plans have now become obligatory to comply with data privacy and safety regulations such as revFADP in Switzerland, GDPR in Europe, and CCPA in California. Although data management plans as well as other documentation are often a sub-section of the experimental protocol, depending on the funding agency, university, or country each of them can be individual documents with different required levels of detail. These documents should be created first and then iterated through revisions to ensure the effectiveness of an experiment before it is run.

The experiment protocol must detail every step of an experiment from all sides. It is important to report both from the participants’ perspective as well as from the process on the experimenters’ side^43^. This ensures that there are no missing steps that may seem obvious from one side but not from the other. Conducting pilot studies and rapid prototyping are crucial tools to ensure the quality of a protocol. Many frameworks use configuration files and experimental flow descriptions that could be used to generate some form of automatic protocol.

#### Infrastructure

The *Infrastructure* pillar addresses the technological foundation that supports experiments, influencing their scalability and efficiency. It encompasses a spectrum of components such as local or remote data processing equipment, content delivery systems, participant equipment, analysis tools, and virtual machines. There are abstraction concepts that have emerged from cloud computing, e.g., an image is a snapshot of an application and its dependencies. a container is a lightweight, portable, and executable instance created from an image with the aim of isolating and running the application consistently across environments and a virtual machine is a virtualized emulation of a physical computer, running an entire operating system and applications independently, in contrast to containers that share the host OS kernel for efficiency and portability. The inclusion of images and containers ensures the reproducibility of software environments, while the research equipment forms the bridge between experimental design and execution. This pillar recognizes the importance of a versatile and reliable technological ecosystem in the experimentation process.

Even though the infrastructure is crucial to conducting an experiment, it is usually informally provisioned and deployed on a per-case basis. Reports usually only state the operating system, the game engine, and sometimes the hardware specifications for data collection. The issue is even starker because a large part of the infrastructure remains unspecified including the data processing, content delivery system, analysis equipment, and researcher equipment. Only unusual configurations produce more detailed documentation, such as multi-user VR^63,67^ but do not provide automation in the form of provisioning or deployment instructions. The rise of IaC offers an opportunity to remove a major hurdle to equitably running VR experiments that to our knowledge so far remain untapped for VR, but see^40,41^ for non-VR approaches.

#### Data collection

At the heart of experimentation lies the meticulous process of *data collection*, embracing both the content of the experiment and the seamless integration of external elements. Experimental content, including tasks, questionnaires, scores, and the overall experiment flow, plays a central role. Simultaneously, the management of data collection interfaces and external content access ensures a streamlined and controlled flow of information. This pillar highlights the importance of precision in capturing diverse data types and managing the interfaces that interact with participants.

Most previous work in VR has focused on the data collection and in particular the experimental content. Second-generation frameworks offered solutions for the technical implementation of the experiment by providing tasks, questionnaires, scores, and state machine (see Fig. 2 Col. 3). Frameworks automate many tedious and repetitive steps that have plagued VR studies by addressing the design-implementation-link problem. Transforming an experiment protocol into a VR experiment is non-trivial. Game engines a priori do not support experimental designs and therefore a mapping from a protocol to an implementation is required. On the one hand, such a mapping can offer fine-grain control, but requires more technical knowledge. On the other hand, a coarse-grain control may not provide sufficient complex features to implement an experimental design.

Thus, there is a trade-off between a generalization of the experiment flow that allows us to execute arbitrarily complexity, e.g.,^68,69^ and simple plug-and-play approaches, e.g.,^62,70,71^. Generalization usually provides researchers with a lot of control and at the same time the responsibility to write accurate experimental flows represented as finite-state machines. Plug-and-play approaches usually provide some prefabricated components that can be placed in the experiment and partially configured with configuration files. A middle ground can be found where experts can develop plug-and-play components based on a generalizable foundation that new users can easily use and with more experience start to adapt to their use case, e.g.,^62,69,72^.

Another important aspect is the Experiment Integration into background services. Here, it needs to be defined what data is to be collected, how the experiment can be managed while it is running, and how external content can be accessed. Data collection requires a concept of the kind of data collected. We can differentiate between virtual sensor data (collected in VR) and physical sensor data (collected by hardware, e.g., HMDs, physiological sensors)^62^. Most frameworks focus on providing a data collection interface for virtual sensors in the virtual environment, disregarding signals from physical sensors, or only looking at virtualized information such as the position and orientation of HMDs and HIDs.

Especially in immersive VR, tracking the user actions such as inputs can be difficult and requires special attention to ensure an accurate reconstruction^73^. Beyond measuring participants' performance in VR, it is often useful to measure their attitudes with surveys^62,74^. subjective measures, and if possible their physiological reaction^43^ to underpin the mechanism investigated in a study beyond self-report and task completion. Keeping only pre-analyzed results such as orientation error is argued to simplify research^72^, but this does not account for calculation errors as occurred in some research projects requiring a redo. Keeping all possible raw data is imperative to produce reproducible and auditable experiments.

Monitoring and managing experiment participants in real time requires a definition and API of how the researcher can interact with the experiment as it happens. Only two frameworks offer out of the box solutions for monitoring^63,75^. Especially, when studying crowd behavior, it may not be enough to conduct single-user experiments, but it becomes necessary to immerse multiple users at once requiring additional infrastructure and experiment integration^63,67^.

#### Data assembly

The *Data Assembly* pillar revolves around the acquisition and preparation of data for analysis. The acquisition phase involves gathering experiment data, infrastructure data, sensor data, and associated metadata. Data-serving APIs facilitate efficient data retrieval. In the data pre- or post-processing phase, anonymization, cleansing, and reduction techniques are applied. This pillar underscores the importance of comprehensive data handling from its origin to its refined form, ensuring the reliability and relevance of the datasets.

The data assembly is mostly secondary in nature in previous research. Many frameworks opt for simple text files, e.g.,^63,72,76^ to reduce the setup requirements for experiments. Only a few frameworks opt for databases, i.e.,^62,69,73,77,78^ because they have more complex use cases in mind. Large amounts of raw data from different sources make text files unwielding and require more effective data assembly. Physical sensor data may be collected at a high frequency^43,76^, which would require special handling that is only integrated into data analysis or as a low frequency approximation during runtime to provide features such as scores. In the best case, the complexity of data assembly can be hidden from the researchers’ view, e.g.,^62^ by only exposing the researcher to data collection and data analysis.

When conducting experiments online, with multiple participants, or at multiple sites, it becomes important to have remote data storage to effectively assemble data from multiple sources, e.g.,^63,69^. Remote storage is orthogonal to questions of data representation in the assembly but requires infrastructure to transmit, store, and access data in the assembly later on. A data serving API is required to organize remote storage and data representation.

Another aspect that has not been addressed by previous research is to transparently wrangle data. Steps of anonymization, cleansing, and reduction should be documented and reproducible. In the context of data reduction, it is possible to resolve, and perform pre-analysis computations that others perform during data collection without maintaining raw data^72^.

#### Data analysis

As experimentation progresses, the *Data Analysis* pillar comes into play, encompassing tasks crucial for making sense of the gathered information. Data preparation involves inspection, validation, fusion, visualization, wrangling, editing, and the extraction of insights. Statistical analyses, simulations, and machine learning-based approaches further unravel the patterns within the data. This pillar recognizes the iterative and multifaceted nature of data analysis, emphasizing the need for thorough exploration and interpretation to draw meaningful conclusions.

The data analysis is usually not considered by second generation frameworks but see^62,72,78,79^. The general move towards pre-registration makes integrating data analysis into frameworks a key requirement to enable researchers to actually go through with pre-registration without a massive overhead. Some frameworks advocate to precompute task scores without keeping the raw data^72^ and present this as an advantage over post-processing by exposing the researcher to less tedious tasks. However, in terms of auditability, having insight into the raw data is an advantage and if data analysis is integrated into the framework, then post-processing can also be largely automated and formally documented.

Effective data processing is crucial to scientific endeavors, but usually, even basic checks are not reported. Ensuring data quality is often not performed or only informally addressed by using robust statistical methods. Data is only visualized based on summary metrics such as walked distance and time spent in spatial cognition experiments. This contrasts with the richness of data that a VR experiment allows to be collected. Providing visualization tools, diagnostic tools for statistical properties, and other things would ensure that the data collected can be used for statistical analysis.

Obtaining insights from data is often performed independent of the experiment and the VR framework. The advantage of keeping data insights within the framework is to tightly connect the analysis with the data collection mechanism and to greatly enhance the reproducibility and reusability. It also allows simulations and machine learning analysis to rely on any collected data.

#### Management

The *Management* pillar takes a holistic view, focusing on overseeing the entire experimental process. Participant acquisition involves sampling, recruiting, payment, and ensuring participants are immersed in the experiment. Monitoring mechanisms track the progress and status of both participants and infrastructure in real-time, sometimes providing live views into ongoing experiments. This pillar stresses the importance of effective oversight to guarantee the integrity, ethical conduct, and success of experiments, from inception to completion.

The management of an experiment is usually not codified in published research and research tools but implicitly performed but see^63,75^. Some parts of the management of the experiments may be reported, such as how participants were selected, but other duties are usually left out. It is important to give special care to management as this neglected component of an experiment is often decisive for the quality of the outcome and the ability to reproduce the work.

### VR Frameworks in the context of ExaC

The pillars used in ExaC provide defined boundaries for modularisation. VR frameworks and specialized software can be used to provide automation code and digital documentation for them. Many existing VR Frameworks already provide support or potential for the automation of many of the common components described within the pillars. We provide a summary of the features of these VR Frameworks through the lens of ExaC and the six pillars in Table 3. Additionally, we provide an in-depth discussion of the VR frameworks through the lens of ExaC in the Supplementary Information Section S1 and a VR-Check list in the Supplementary Information Section S2.

**Table 3:** Comparison of current VR frameworks since 2015.

Framework	Platform	Experiments as Code																
		Doc.	Infrastructure				Data Collection				Data Assembly		Data Analysis		Managem.		Access	
		Protocol^†^	Multi-user Server	Immersive VR	Provisioning^†^	Deployment^†^	Prefab. tasks	Task config. files	Game engine code	Questionnaires	Remote Storage	Database	Analysis	Replay	Participants	Monitoring	github/bitbucket	Unity package
AGENT^68^	Any							$\checkmark$	$\checkmark$									
bmlTUX^75^	Unity							$\checkmark$	$\checkmark$							$\checkmark$	$\checkmark$	
EVE^62^	Unity			*			$\checkmark$	*	$\checkmark$	$\checkmark$		$\checkmark$	$\checkmark$	$\checkmark$			$\checkmark$	
DeFINE^77^	Unity			$\checkmark$				$\checkmark$	$\checkmark$	$\checkmark$	$\checkmark$	$\checkmark$					$\checkmark$	
Landmarks^72^	Unity			$\checkmark$			$\checkmark$	$\checkmark$	$\checkmark$	$\checkmark$			*				$\checkmark$	$\checkmark$
NVR-DeSciL^63^	Unity		$\checkmark$						$\checkmark$		$\checkmark$				$\checkmark$	$\checkmark$		
OpenMaze^80^	Unity			*				$\checkmark$									$\checkmark$	
Toggle Toolkit^71^	Unity			*			*										$\checkmark$	
VO^73^	Unity			$\checkmark$					$\checkmark$		$\checkmark$	$\checkmark$		$\checkmark$				
VRate^81^	Unity			$\checkmark$				$\checkmark$		$\checkmark$								
VREVAL^79^	Unreal			$\checkmark$			$\checkmark$			$\checkmark$			$\checkmark$					-
VREX^70^	Unity			$\checkmark$			*	*	*									
VR-Rides^78^	Unity			$\checkmark$					$\checkmark$			$\checkmark$	*				$\checkmark$	
USE^76^	Unity							$\checkmark$									$\checkmark$	
UXF^69^	Unity			$\checkmark$				$\checkmark$	$\checkmark$		$\checkmark$	$\checkmark$					$\checkmark$	$\checkmark$

The features are grouped by correspondence to the six pillars taxonomy of the *ExaC* paradigm. Features are evaluated on the description of the frameworks in their accompanying paper. We also report the availability of the frameworks. The development of *ExaCT* aims to address gaps found in current VR frameworks to better align them with the specific requirements of each of the six experimental pillars identified.

$\checkmark$: Feature, *: Partial Feature, -: Not possible, ^†^: Never implemented.

In the early 2000s, a first set of VR frameworks^82–86^ focused on simplifying the technical setup. However, these frameworks were rapidly eclipsed by game engines (i.e., Unity and Unreal) and became quickly obsolete. Over the last five years, a second generation of VR frameworks^62,63,68,69,71,72,75,76,78–80^ has put the experiment design at the core of the framework. Technical issues such as rendering, physics, and specialized hardware have been delegated to the underlying game engines such as Unity or Unreal and third-party libraries to integrate Human Interface Devices (HIDs; e.g. Oculus Touch, 3D mice).

The second generation of VR frameworks provides better support to the different components required for experimentation. However, there is still a considerable gap in terms of hardware provisioning and deployment, support for data analysis, multi-user setups, and participant management and monitoring. To bridge these gaps, we envision a third generation of VR frameworks that either natively support all six pillars of ExaC or that are modularly designed to better integrate with specialized tools that cover them. This third generation would extend their focus from experimental design towards reproducibility, auditability, debuggability, reusability, and scalability. General frameworks for behavioral experimentation recently started focusing on reproducibility^40,41^. However, we believe that for the future development of VR frameworks with reproducibility as the cornerstone, a robust definition of the underlying paradigm to achieve and document reproducibility is required. Hence this paper proposes and defines ExaC as the underlying paradigm and proposes a taxonomy to support its modular development.

### ExaC implementation

To demonstrate the ExaC paradigm, we provide a proof-of-concept automation code covering each of the six pillars of experimentation (see Fig. 2). For this, we used open source tools (i.e., Terraform, jupyter-lab, and Docker) coupled with a minimal ad-hoc framework we named SimpleVR written in C#/Unity, Python, JavaScript and Golang. This section goes through each of the pillars and provides a concise description of our automation code and technologies covering the respective pillars. Finally, we present an example workflow. Although the provided implementation of ExaC has been used for several of our experiments, it should be considered as a minimal working example to demonstrate the concept and not a mature general-purpose code base. One of the added advantages of this modular design is the potential evolutive improvement. Consequently, successful ExaC code bases and code components will be replicated, modified, and improved.

In our implementation of the ExaC paradigm, we distinguish between three layers (see Fig. 5, A). The top layer consists of the software stack that describes what the experiment is (see Fig. 5, A3–A6). The middle layer consists of the software stack that describes how the experiment is run (see Fig. 5, A2). The bottom layer consists of digital documentation management (see Fig. 5, A1). The distinction between the stacks is similar to the differentiation between a dev-stack and a devops-stack in software service development. The dev-stack refers to a *dev*eloper and the software they use to produce a program whereas the devops-stack refers to the interplay between *dev*elopment and *op*eration*s* that are needed to make a program run.

In the top layer, we provide an implementation of the four pillars with our proof-of-concept framework SimpleVR, analogous to the dev-stack. In the middle layer, we provide software defining run-time environments, automation, provisioning, and deployment, analogous to the devops-stack. Our ExaC implementation is the composite of these three layers, which we refer to as SimpleExaCT^87^. The middle layer of the ExaC implementation supports Preproducibility by automating the deployment through cloud computing concepts, namely EaC and IaC. We containerized the top layer services using EaC (i.e., a Docker image). The middle layer provisions the infrastructure and deploys the containerized services using IaC (i.e., Terraform).

Since the system is meant to support reproducible experiments, we pay particular attention to the top layer that contains the experiment-defining code. To this end, we address its design considerations in light of our case study needs, specifically remote VR experimentation^88^. For the most part, conducting VR studies has been the privilege of well-funded institutions because of the prohibitive costs of large-scale experimental data collection. We address this issue with a minimalistic ad-hoc VR framework, SimpleVR, by reducing the computational cost of large-scale data collection. In traditional lab-based VR experiments, the researchers need to provide the computational power to run each of the VR experimental trials. This is done through a central server or offloaded to the lab computers.

In contrast, SimpleVR offloads the VR computations to the participants’ browser and requires the researchers to provide only the infrastructure needed to capture the transferred data. This is supported by new web browsers that provide local browser storage, service workers, access to 3D rendering capabilities, in-browser databases, and the ability to execute code with low overhead through web-assembly. It needs to be noted that the purpose of this paper is not to present the development of a feature rich VR framework nor to discuss the pros and cons of online or in-lab experiments. Instead, we focus on the novelty of the ExaC paradigm. Even-though ExaC is the paradigm, the concept can be used for online or in-lab experiments. The minimal framework (i.e., SimpleVR) presented in the proof of concept was developed to bring the experiment to the browser and minimally covers the top layer in the six pillar taxonomy.

SimpleVR represents the top layer of the ExaC code Base (see Fig. 5, A3–A6). When deployed, the instances of the four composable component groups serve a number of purposes. The first component group (see Fig. 5, A3) defines the data collection software which is displayed in the client browser (see Fig. 5, E). Trials of independent human participants run within their web browsers to collect data. The second component group (see Fig. 5, A4) defines the data assembly service which is deployed in the cloud (see Fig. 5, D). This component group provides an API to the first component detailing how to collect and store the data. The third component (see Fig. 5, A5) defines the data analysis service that allows the processing of data and gaining insights (see Fig. 5, B). This component group interacts with the data assembly. The fourth component group (see Fig. 5, A6) defines the data management service that allows the researcher to monitor and manage the experiment (see Fig. 5, B & C) and interacts with all of the three previous component groups. Once the data collection and data assembly services are ready, the experiment deployment infrastructure distributes the data collection software to the participants.

The SimpleExaCT code base consists of the digital documentation, the IaC and EaC technologies, and SimpleVR. We refer to it as the *Base* (see Fig. 5, A).

This section provides details on the components of ExaC implementation of a simple wayfinding experiment in the field of Cognitive Science. All components are provided in the GitHub repository: https://github.com/leaguilar/SimpleExaCT. To handle the synchrony between the components’ versions we used the ‘mono repo’ approach, where every component is contained in a subfolder of the same repository. In other words, this single repository holds all the experiment’s code, e.g., documentation, provision, deployment, data collection, data assembly, data analysis, and experiment management code. Additionally, every component uses its specific versioning pinning mechanism, e.g., Terraform’s version pinning, and Docker image definition.

#### Documentation—A1

The digital documentation for this example implementation consists of the necessary meta-information. It includes traditional software metadata, the README, AUTHORS, and LICENSE files along with the experiment specific documentation such as the experiment protocol. Incepting a good protocol is still more of an art but certain guidelines are available such as the VR-Check^89^. While it focuses on developing neuroscience training in VR, it also offers an overview of key attributes to consider when drafting any experiment protocol (see the Supplementary Information Section S2). Apart from the traditional software requirement documentation (e.g., package.json, requirements.txt, and Pipfile) and the traditional experiment documentation (e.g., the protocol) we include a service documentation file services.json, denoting the requirements in terms of external services (i.e., abstraction layer services, and infrastructure services) required for the experiment (e.g., MTurk, AWS).

#### Infrastructure—A2

SimpleExaCT defines the required run-time environments with Docker (i.e. EaC; see Fig. 5, A2) and automates the provisioning and deployment of the required experimental infrastructure through *Terraform* scripts (i.e. IaC; see Fig. 5, A2). The script creates an S3 bucket and uploads the static content, reserves the computing infrastructure for the data assembly service, and deploys it. All this is initiated directly from the researchers’ computer. Once the data collection has finished, the *Terraform* scripts automate the stop and release of all cloud resources used in the experiment.

#### Data collection—A3

The participants join the experiment with their web browser. In practice, a link is provided and the experimental data collection client is loaded as any other web-page. As participants visit this link, the experiment goes through 3 phases: the *Onboarding* of participants, the participants’ engagement in the *Experiment* trial and finally their *Offboarding* (see Fig. 5, E). SimpleVR contains scripts and templates that instrument this data collection procedure (see Fig. 5, A3).

In the onboarding, we perform two checks to ensure that both Unity can be started and that it runs at a reasonable frame rate. We use Unity’s support of web-assembly with WebGL as “build” target to offload the VR computations to the web-browser. To determine if the web-assembly module can be loaded, the browser compatibility and rendering capabilities are detected through Unity. The prospective quality of rendering is tested using the test suite of mapboxgl (a JavaScript library). If the requirements are fulfilled, the experiment content is loaded. The Unity-based data collection client receives the parameters to instantiate the experiments’ content along with authentication for participants’ payment.

In the experiment, the specific treatment tested in this trial is instantiated for data collection, and the content is rendered in the participants’ browser (see Fig. 5, E). The whole experimental trial is distributed as static content. For example, in the presented case study through an AWS S3 Bucket. We used Unity to render, control, record, and transfer movement data from the virtual environment on the participants’ computers. The collected experimental data is sent back to the data assembly service continuously throughout the trial. The client can send different types of messages to the data assembly service to assemble, store, and make it available to the researcher. For the simplicity of the demonstration, we implemented two main types, which are event messages and trajectory messages. Event messages provide metadata about a specific event (e.g., agreeing on the consent form, or completing a specific task). Trajectories are recorded by logging participants’ positions and orientations with varying time resolutions. Trajectories represent heavier data transfers and thus are segmented into packages. Data is transferred in the background while the participants complete the tasks (i.e., in 4.3Kb chunks) to the data collection service and stored in a secure cloud storage.

In the offboarding, once the participants have completed the trial, a response code is calculated by the data collection client to signal the conclusion of the experiment. The code is based on an implementation of the Salted Challenge Response Authentication Mechanism (SCRAM)^90^. Here, a challenge code is generated and combined with a researcher-provided authentication called “salt”. This delivers the signal to external services like MTurk that participants have completed their tasks and that these services can transfer the payment.

#### Data assembly—A4

The Data Assembly service provides the backend for data collection and securely stores and makes the data available to the researcher. This service (see Fig. 5, A4) has been built as a lightweight go program due to the language’s embedded concurrency features. To deploy the Data Assembly service, we containerize it using Docker. Heavy traffic experiments may require the scaling of the data collection service and running it as part of a Kubernetes cluster. This enables having detailed control over the load balancer and reverse proxies for load sharing.

For the data transmission, the service receives the POST messages of the clients and stores them in the service’s local persistent storage. The transmitted messages provide header and tail identifier messages. The tails provide a checksum over the transferred data to ensure the correct reconstruction of the transferred data once all packages/chunks have been received. Once the tail packet is received, the data for a specific trial is reconstructed and uploaded to a secure cloud storage (in the case study we use an AWS S3 bucket). The data is transferred as JSONs for its reconstruction. The reconstructed data is assembled into CSV files and uploaded into a cloud storage solution (e.g., AWS S3 bucket).

Data transmission between the data collection service and the data assembly service is secured to prevent mishandling. It takes advantage of HTTPS (HTTP over TLS) enabling the encrypted transport of the data. It has to be noted that modern web browsers will refuse to establish a connection over HTTPS with self-made SSL certificates. If no SSL certificates are available, researchers will be forced to revert back to insecure transmission and protect the data transfers themselves, i.e. encrypt the data instead of the channel.

#### Data analysis—A5

To exemplify the data analysis, a Python Jupyter notebook with a minimal working example for our case study’s analysis is provided. During the deployment of SimpleExaCT, a container with Jupyter-Lab on the local researcher's computer is run to provide an interface to the bundled notebooks (see Fig. 5, A5 & B ). This component is well-qualified to serve as the basis for pre-registration for an analysis plan.

#### Management—A6

The experiment is scaffolded with cloud infrastructure. It can be served to anyone with a capable web browser through a link to the static content and the corresponding *Experiment* (see Fig. 5, E). To address the difficulty of recruiting and securely rewarding the participants, we deployed the experiment through Amazon Mechanical Turk (MTurk).

To monitor and manage both the hardware and participants, we include two further Python Jupyter notebooks, (see Fig. 5, A6). It has to be noted that monitoring and management should not be interpreted as *surveillance*. It is a privacy-preserving, consensual, and non-intrusive check of the state of the experiment and the health of the hardware. We reuse the same dockerized local Jupyter-Lab instance mentioned in the data analysis to provide access to these notebooks (see Fig. 5, B). The participant monitoring and management notebooks interact with Amazon Mechanical Turk’s API and serve to link our experiment with specific challenge codes and the correct treatment assignment, once the experiment is completed. Additionally, it verifies the correctness of the responses to the challenges and rewards the participants. Through the hardware management notebook, we monitor the state of the cloud infrastructure. The current proof-of-concept implementation triggers an alarm sound when the Data Assembly service is unreachable.

### Demonstrative case study design

We selected a desktop VR experiment in HBI that does not require specialized equipment beyond a capable computer and a browser and is robust under a wide range of uncontrolled variations, e.g., variations in screen sizes, or distance to the screen. This enabled us to easily showcase the use of remote data collection and demonstrate the scaling of participants. This experiment was carried out in April 2020 in three sessions, during the COVID-19 pandemic and lockdowns,^33^.

The data collection was spread out across days to obtain enough participants according to the power analysis. The three sessions were instantiated from scratch and can be understood as three independent experiments. The third session did not collect data on all treatments because only some treatments required more data to fulfill a preliminary power analysis. Hence, the results presented in this paper include only a comparison between the two full instantiations, sessions 1 and 2. For demonstration purposes and to avoid conflict with the detailed publications of the experiment, here we refer in general terms to: (1) the control and applied treatments which were architectural variations on a building, and (2) the dependent variable used to measure the participant’s performance. Details on the experiment and further behavioral analysis of the MTurk participant data can be found here^33^. Details on the validation of an Agent-Based Model (ABM) using this data can be found here^58^.

Participants were recruited online through MTurk Human Intelligence Tasks (HITs). Participants were filtered according to the external requirements of informed consent, technical ability to display the experiment, and completion of the experiment. We only used data from participants whose web browser was deemed capable of displaying the experiment, who agreed on the consent screen, and who completed the task with the correct response to the challenge. 149 participants fulfilled these criteria. Participants’ ages ranged between 18 years and 59 years. The mean age was 33.7 years with a Standard Deviation of 6.8 years. The methods and experiment described in this section were performed in accordance with the relevant guidelines and regulations following the approval of the study by ETH Zurich’s Research Ethics Committee (2020-N-24). Two inclusion criteria for the study were English proficiency, and corrected-to-normal or normal vision. Color-blind individuals were excluded from participating in the study. All participants signed an informed consent form. The average time taken to complete the experiment was 20 minutes and the average monetary compensation was 4.5 USD. If participants completed the experiment in less than 20 minutes, they received a 1 USD bonus. The average compensation was 5.4 USD.

*Experimental protocol*: The protocol of the experiment can be found in the SimpleExaCT repository,^87^ and a complete description of the demonstrative experiment content, the experimental protocol, materials, and data analysis can be found at^58^. Overall, the experiment tested how changes in the configuration of buildings may affect human navigation performance.

Figure 5 shows how ExaC was used to deploy the experiment. An abstraction layer (Fig. 5, C) was in interfacing with the participant procurement service. For logistical reasons, we opted for the DeSciL and MTurk as specific service providers in charge of communication with both the researcher and with the study participants. Nonetheless, we provide scripts to interface with MTurk directly within the SimpleExaCT implementation. Communication with participants mainly consisted of deploying the HITs for them to perform the experiments.

Participants had a first person perspective of the virtual scene in each building. Participants’ origin was set to the same location across the three building groups (see Fig. 5, E). Navigation was performed using a keyboard and mouse. Participants had to digitally sign a consent form and complete the onboarding before the experimental trials and the offboarding afterward. The onboarding ensured the correct assignment to one of the three building conditions and the offboarding the appropriate reward. The experiment itself mainly consisted of two parts, a training phase and 6 navigation trials. The mean duration of the training phase was 5 min. During training, participants learned how to move in the virtual environment using a mouse and keyboard. This is a common procedure to ensure that participants are familiar with the controls to reduce adverse effects^91,92^. Upon successful completion of the practice scene, participants had to complete 6 trials in which they were asked to find a specific destination inside the building.

The participants’ positions and orientations were recorded every frame (roughly every 0.02 s) using a cloud infrastructure to deploy and retrieve participants’ data (see Fig. 5, C and D). The research team (see Fig. 5, B) was able to manage and monitor participants, hardware and software status from their computers. The abstraction services provided interactions with the management of participants (see Fig. 5, B and C). The cloud infrastructure provided interactions with both the deployment of the experiment content and access to the collected data (see Fig.5, B and D). Using the analysis scripts, the research team was able to visualize and analyze the data collected immediately after the experiment was concluded (see Fig. 5, B).

### Informed consent

The research with human participants was approved by the Research Ethics Committee of ETH Zürich (2020-N-24). The participants were informed on the study goal and gave informed consent and accepted the publication of appropriately anonymised data.

### Supplementary Information

Supplementary Information.

## Data Availability

The workflow required to reproduce the experiment is quite simple. The instructions are provided in the README of the github repository^87^. The checklist is summarised here: 1. Fill in or customise the variables in the Terraform script to access cloud services (e.g., AWS, MTurk etc). 2. Provision and deploy experiment infrastructure: # terraform init, and # terraform apply. 3. Release experiment trials (HITs) for participants to complete through the provided MTurk’s scripts. 4. Manage and Monitor the experiment through the management scripts. 5. Download your data and analysis results.
