# Supplementary material for: Experiments as Code and its application to VR studies in human-building interaction
Source: Sci Rep. 2024 Apr 30;14:9883. doi: 10.1038/s41598-024-60791-3 (PMC11061313; doi:10.1038/s41598-024-60791-3)
Supplement: Supplementary file 1 — Supplementary Information. [file 41598_2024_60791_MOESM1_ESM.pdf]

Supplementary Information - *Experiments as Code* and its Application to VR Studies in Human-Building Interaction

Leonel Aguilar[1,2*+], Michal Gath-Morad[3,1*], Jascha Grübel[1,5,6,7,8,9*], Jasper Ermatinger[1], Hantao Zhao[1], Stefan Wehrli[10], Robert W. Sumner[5], Ce Zhang[2], Dirk Helbing[10,11], and Christoph Hölscher[1,10]

[1]Chair of Cognitive Science, ETH Zürich, Switzerland
[2]Data Science, Systems and Services Group, ETH Zürich, Switzerland
[3]Cambridge Cognitive Architecture, University of Cambridge, UK
[5]Geo-information Science and Remote Sensing Laboratory, Wageningen University, The Netherlands
[6]Game Technology Centre, ETH Zürich, Switzerland
[7]Visual Computing Group, Harvard University
[8]Center for Sustainable Future Mobility, ETH Zürich, Switzerland
[9]Geoinformation Engineering Group, ETH Zürich, Switzerland
[10]Decision Science Laboratory, ETH Zürich
[11]Chair of Computational Social Science, ETH Zr̈ich
[*]Equal contribution
[+]Corresponding author: leonel.aguilar@gess.ethz.ch

S1 Frameworks for experimentation in VR through the lens of ExaC

In this section, we provide a more detailed discussion of the frameworks mentioned in Table 3 of the main text, highlighting their individual contributions to the overall development of VR experiments. This review of frameworks is an integral part of our iterative process to examine how various elements of an experiment are currently being documented, automated, and interfaced within existing experimentation tools. By doing so, we were able to assess whether the proposed six-pillar taxonomy adequately covers the existing automated aspects of experiments. Throughout this review, we keep a focus on the six pillars of ExaC (refer to Figure 2 in the main text) to establish a connection between the potential for automation.

The Experiments in Virtual Environments (EVE) framework[1] introduced a focus on automating data collection, assembly and analysis. An experiment protocol is implemented using prefabricated (prefab) templates or creating new scenes with tasks and annotations on which data to store. The design-implementation-link problem for novices is addressed with prefabs but experts are required to create new solutions. At runtime, the framework manages the participant sessions and data assembly based on configuration files. This framework also offers questionnaires and data analysis in an R package to bring together all data associated with an experiment in a single place and simplify difficult steps in data assembly and data analysis. This setup allows for rapid prototyping and piloting including the analysis of the experiment, which is crucial for preregistration. The framework is open-source and has been regularly extended with new templates based on studies conducted over the years[2–5] and extended to run on all major operating systems.

The Virtual Reality Experiments (VREX)[6] was developed at the same time as EVE. Plug-and-play design of experiments is fully achieved by providing a stand-alone software to edit the experiments based on Unity. This limits the experiments to predefined tasks but in a fully configurable indoor environment. Other interesting features are configurations for spatial audio and different locomotion systems. The data collection is a rudimentary data logging to files of fixed variables. The authors also provide the actual unity project which would allow to create new tasks to expand the stand-alone version.

Expanding on the idea to automate difficult steps in experimental design in VR, the Automatic Generation of Experimental Protocol Run Time (AGENT)[7] uses Domain-Specific Language (DSL) to facilitate the description of data collection. This approach is meant to reduce even further the workload on the researcher by generalizing typical experimental protocols. This approach works well in organizing the experimental protocol and generating the code to execute it. However, it requires the researcher to link the generated code to objects in their scenes (design-implementation-link problem). Ultimately, the DSL cannot overcome the necessity to have in-depth technical knowledge of the game engine. AGENT also presumes the researcher to have a high-quality protocol in order to automatically generate the experiment code. As an external library, AGENT could be integrated with any game engine but only provides Unity API.

The framework VRate[8] focuses on providing questionnaires in VR. Questionnaires were implemented to work in immersive

VR and are created from JavaScript Object Notations (JSON) and stored in comma separated files (csv). There is no support for the implemention of experiments beyond questionnaires.

Previous frameworks have focused on tailored environments to study general phenomena. The VREVAL framework[9] focuses on automating experiment environments by integrating building information models (BIM). Here, BIMs are combined with questionnaires, navigation tasks, and annotation tasks to conduct research that can be evaluated with external BIM software. It is one of the few frameworks, to our knowledge, that uses the Unreal game engine.

Another interesting angle of research that is enabled by running VR studies is to understand how people react in crowds both under normal and emergency conditions. The Networked Virtual Reality for the Decision Science Laboratory (NVR-DeSciL)[10] focuses on studying crowd dynamics in VR and is a networked solution to enable multiple participants in a single virtual environment at the same time. An authoritative server architecture is used to provide the same experience to all participants. Here, all user inputs are sent to the server and an update is produced for all participants at once. The framework is tailored to the Decision Science Laboratory (DeSciL) at ETH Zürich but offers an interesting approach how to automate VR for multi-participant experiments.

Another framework that focuses on the execution of the experiment protocol is the Unity Experiment Framework (UXF)[11]. Instead of using a DSL like AGENT, they programmatically separate the what from the how to address the design-implementation-link problem. Here, the framework provides a generalization of the experiment flow in the form of sessions and trials and researchers must register with the sessions and trials what should happen in C# code. In a second step, the researcher must input how by writing C# code snippets on how a trial within a session is run. UXF provides many prebuild code snippets to design classic experiments. While the setup does not quite follow a classical "___ as Code" approach in the form of a configuration file, it can be considered one as the C# code is essentially reading the configuration and executing it.

The Unified Suite for Experiments (USE)[12] focuses on neuroscience application by providing hard- and software to automate synchronizing data with high temporal precision in local labs. The frame rate as well as transmission delays produce serious issues for brain-scanning where the exact stimulus must be determined. USE introduces the SyncBox as a hardware setup that measures the current frame on the screen and aligns it ex-post with the high-frequency data from brain-scanners and other hardware. The framework also provides a nested hierarchy and a user-defined experimental flows to create experiments tasks, providing yet another attempt how to solve the design-implementation-link problem. Additionally, the framework is able to automate interactions for artificial and non-human users.

Another attempt at the design-implementation-link problem has been taken by the framework Biomotion Lab Toolkit for Unity Experiments (bmlTUX)[13]. It is a small framework that focuses on organizing participants into a factorial design with dependent and independent variables, providing a nested design for experiments but ultimately requires the coding of the trial. It compares to UXF or AGENT in providing a user interface for the factorial design instead of coding it in C# or a DSL.

OpenMaze[14] focuses on a particular type of navigation experiments and tries to provide automations with maximal flexibility and low complexity. Configuration files are used to define the relations between goals, landmarks, and enclosures. Here, the design-implementation-link problem is solved by restricting the input space and providing pre-configured implementations.

Data assembly is rarely the focus of a framework. Virtual Observations[15] focuses on the reconstruction of user actions. In particular, immersive VR user input requires special care as handheld controllers spatially located in the virtual scene produce a large amount of data that critically defines the participants' action in the scene. The framework advances replay and monitoring functionality but does not provide features for experimental design beyond recording the necessary data. This framework is mostly orthogonal to other frameworks and could be used in parallel but without specific integration would duplicate data recording features. Nonetheless, the more fine-grained approach to user input in immersive VR is a valuable addition to automation practices.

The Landmarks framework[16] focuses on data collection, assembly and analysis like EVE but sets different focuses. It also includes different immersions (HMD, Desktop, etc), the timeline of an experiment (a nested hierarchy) including tasks, the environment and the data collection with log-files. A unity package is provided easing the access to developing an experiment.

VR-Rides[17] is not a classical experiment framework but focuses on exercise games (exergames) where users physically move with either a pedaling device or a treadmill through a Google Street View (GSV). It provides automation for hardware and GSV. Interestingly, the framework provides a user-study module that allows to assign participants to conditions, as well as a database allowing to implement experiments.

The Toggle Toolkit[18] provides an interesting approach to resolve the design-implementation-link problem. It introduces *trigger* as a design concept to transfer unity features to experimental design paradigms (autonomous trigger, key trigger, timer trigger, collider trigger, collider-key trigger, distance trigger). The triggers can be chained producing something similar to a experimental flow but embedded in the scene. While expressing complex task designs may be tedious, it can be performed without writing code providing an effective solution to the design-implementation-link problem.

The framework Delayed Feedback based Immersive Navigation Environment (DeFINE) for Studying Goal-Directed Human Navigation is an extension of UXF with a focus on the stimulus–response–feedback architecture for navigation experiments. In

particular, the feedback is an explicit part of the framework where other frameworks require the researcher to develop these features themselves. The feedback is presented in the form of a leader board to encourage participants to perform better. The framework also provides researchers with a set of locomotion methods including teleport, arm swing, head-bob and physical walking. Lastly, it is also one of the few frameworks to provide immersed questionnaires to maintain immersion for participants.

S2 VR-Check for general VR experiment

The VR-Check[19] has been developed to underpin the development of VR software for medical use with the main purpose to retrain humans after severe physical or neuronal trauma. While the original checklist is thus very domain specific, we generated a more general notion to help improve the development of an experimental protocol, experimental documentation and most of all, experimental design. We rephrase the checklist as questions to make them more accessible to the researcher using them.

1. *Domain specificity*: Can the task in question produce a valid measurement for the research question?
2. *Ecological validity*: Does the measurement generalize to phenomena in the real world?
3. *Technical feasibility*: Can the task be measured with the given technical setup?
4. *User feasibility*: Can the users complete the task given the task difficulty and the technical implementation (e.g. motion sickness)?
5. *User motivation*: Does the participant want to solve the task to the best of his ability?
6. *Task adaptability*: How fine-grained can the difficulty of the task be adjusted and can variations of the task be easily created?
7. *Performance quantification*: How suitable is the measurement to detect variation in task performance?
8. *Immersive capacities*: How does immersion (ability to perform the task based on hardware) compare to presence (the sense of being there influences task performance/user engagement) in the experimental design. For a detailed discussion see[20–22]?
9. *Training feasibility*: How often can the task be repeated to become solvable and how often has the task to be repeated to become trivial?
10. *Predictable pitfalls*: Where does the experimental design invite participants to behave other than intended?

S3 Debuggability and Reusability examples

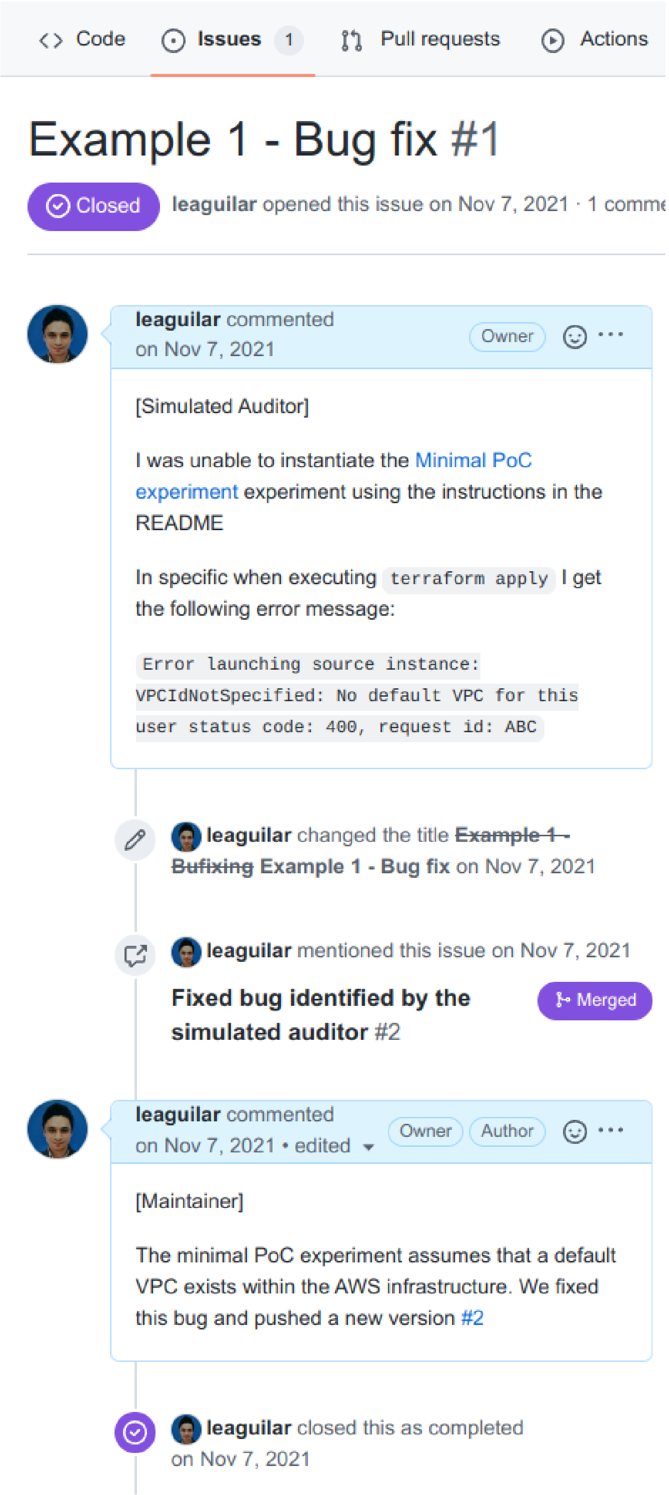

Figure S1. Screenshot of the simulated Auditing and Debugging interaction. link: https://github.com/leaguilar/SimpleExaCT/issues/1

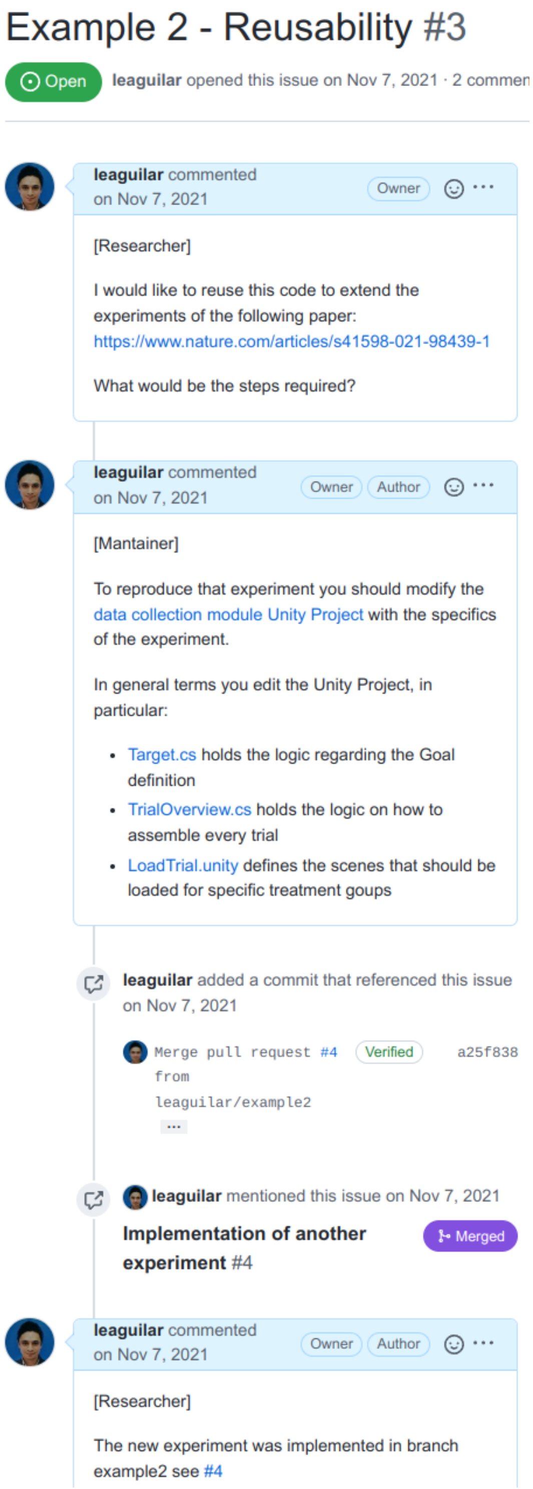

Figure S2. Screenshot of the reuse of an existing experiment. link: https://github.com/leaguilar/SimpleExaCT/issues/3

References

1. Grübel, J. *et al.* Eve: A framework for experiments in virtual environments. In *Spatial Cognition X*, 159–176 (Springer, 2016).
2. Weibel, R. P. *et al.* Virtual reality experiments with physiological measures. *JoVE (Journal Vis. Exp.* **1**, e58318 (2018).
3. Hackman, D. A. *et al.* Neighborhood environments influence emotion and physiological reactivity. *Sci. reports* **9**, 1–11 (2019).
4. Fischer, J., Wissen Hayek, U., Galleguillos Torres, M., Weibel, B. & Grêt-Regamey, A. Investigating effects of animated 3d point cloud simulations on emotional responses. *J. Digit. Landsc. Archit.* **5**, 295–304 (2020).
5. Spielhofer, R. *et al.* Physiological and behavioral reactions to renewable energy systems in various landscape types. *Renew. Sustain. Energy Rev.* **135**, 110410 (2020).
6. Vasser, M. *et al.* Vrex: an open-source toolbox for creating 3d virtual reality experiments. *BMC psychology* **5**, 1–8 (2017).
7. Moulec, G. L., Argelaguet, F., Gouranton, V., Blouin, A. & Arnaldi, B. Agent: automatic generation of experimental protocol runtime. In *Proceedings of the 23rd ACM Symposium on Virtual Reality Software and Technology*, 1–10 (2017).
8. Regal, G., Schatz, R., Schrammel, J. & Suette, S. Vrate: a unity3d asset for integrating subjective assessment questionnaires in virtual environments. In *2018 Tenth International Conference on Quality of Multimedia Experience (QoMEX)*, 1–3 (IEEE, 2018).
9. Schneider, S., Kuliga, S., Weiser, R., Kammler, O. & Fuchkina, E. Vreval-a bim-based framework for user-centered evaluation of complex buildings in virtual environments. In *Proceedings of the 36th eCAADe Conference* (CUMINCAD, 2018).
10. Zhao, H. *et al.* A networked desktop virtual reality setup for decision science and navigation experiments with multiple participants. *JoVE (Journal Vis. Exp.* **1**, e58155 (2018).
11. Brookes, J., Warburton, M., Alghadier, M., Mon-Williams, M. & Mushtaq, F. Studying human behavior with virtual reality: The unity experiment framework. *Behav. research methods* 1–9 (2019).
12. Watson, M. R., Voloh, B., Thomas, C., Hasan, A. & Womelsdorf, T. Use: An integrative suite for temporally-precise psychophysical experiments in virtual environments for human, nonhuman, and artificially intelligent agents. *J. neuroscience methods* **326**, 108374 (2019).
13. Bebko, A. O. & Troje, N. F. bmltux: Design and control of experiments in virtual reality and beyond. *i-Perception* **11**, 2041669520938400 (2020).
14. Alsbury-Nealy, K. *et al.* Openmaze: An open-source toolbox for creating virtual environment experiments (2020).
15. Howie, S. & Gilardi, M. Virtual observations: a software tool for contextual observation and assessment of user's actions in virtual reality. *Virtual Real.* 1–14 (2020).
16. Starrett, M. J. *et al.* Landmarks: A solution for spatial navigation and memory experiments in virtual reality. *Behav. Res. Methods* 1–14 (2020).
17. Wang, Y., Ijaz, K., Yuan, D. & Calvo, R. A. Vr-rides: An object-oriented application framework for immersive virtual reality exergames. *Software: Pract. Exp.* **50**, 1305–1324 (2020).
18. Ugwitz, P., Šašinková, A., Šašinka, Č., Stachoň, Z. & Juřík, V. Toggle toolkit: A tool for conducting experiments in unity virtual environments. *Behav. research methods* 1–11 (2021).
19. Krohn, S. *et al.* Multidimensional evaluation of virtual reality paradigms in clinical neuropsychology: Application of the vr-check framework. *J. medical Internet research* **22**, e16724 (2020).
20. Slater, M. Place illusion and plausibility can lead to realistic behaviour in immersive virtual environments. *Philos. Transactions Royal Soc. B: Biol. Sci.* **364**, 3549–3557 (2009).
21. Slater, M. & Sanchez-Vives, M. V. Enhancing our lives with immersive virtual reality. *Front. Robotics AI* **3**, 74 (2016).
22. Slater, M. Immersion and the illusion of presence in virtual reality. *Br. J. Psychol.* **109**, 431–433 (2018).
